# Beyond population size: Whole-genome data reveal bottleneck legacies in the peninsular Italian wolf

**DOI:** 10.1093/jhered/esae041

**Published:** 2024-08-27

**Authors:** Daniele Battilani, Roberta Gargiulo, Romolo Caniglia, Elena Fabbri, Jazmín Ramos- Madrigal, Claudia Fontsere, Marta Maria Ciucani, Shyam Gopalakrishnan, Matteo Girardi, Ilaria Fracasso, Matteo Mastroiaco, Paolo Ciucci, Cristiano Vernesi

**Affiliations:** Department of Biology and Biotechnologies “Charles Darwin”, Università di Roma La Sapienza, Roma, Italy; Area per la Genetica della Conservazione, ISPRA, Ozzano dell’Emilia Bologna, Italy; Center for Evolutionary Hologenomics, The Globe Institute, University of Copenhagen, Copenhagen, Denmark; Ecosystem Stewardship, Royal Botanical Gardens, Kew, United Kingdom; Area per la Genetica della Conservazione, ISPRA, Ozzano dell’Emilia Bologna, Italy; Area per la Genetica della Conservazione, ISPRA, Ozzano dell’Emilia Bologna, Italy; Center for Evolutionary Hologenomics, The Globe Institute, University of Copenhagen, Copenhagen, Denmark; Center for Evolutionary Hologenomics, The Globe Institute, University of Copenhagen, Copenhagen, Denmark; Center for Evolutionary Hologenomics, The Globe Institute, University of Copenhagen, Copenhagen, Denmark; Center for Evolutionary Hologenomics, The Globe Institute, University of Copenhagen, Copenhagen, Denmark; Research and Innovation Centre-Fondazione Edmund Mach, S. Michele all’Adige, Italy; Research and Innovation Centre-Fondazione Edmund Mach, S. Michele all’Adige, Italy; Department of Biology and Biotechnologies “Charles Darwin”, Università di Roma La Sapienza, Roma, Italy; Department of Biology and Biotechnologies “Charles Darwin”, Università di Roma La Sapienza, Roma, Italy; Research and Innovation Centre-Fondazione Edmund Mach, S. Michele all’Adige, Italy

**Keywords:** *Canis lupus*, conservation genomics, genetic diversity, genetic load, inbreeding, *N*_e_ estimation

## Abstract

Preserving genetic diversity and adaptive potential while avoiding inbreeding depression is crucial for the long-term conservation of natural populations. Despite demographic increases, traces of past bottleneck events at the genomic level should be carefully considered for population management. From this perspective, the peninsular Italian wolf is a paradigmatic case. After being on the brink of extinction in the late 1960s, peninsular Italian wolves rebounded and recolonized most of the peninsula aided by conservation measures, including habitat and legal protection. Notwithstanding their demographic recovery, a comprehensive understanding of the genomic consequences of the historical bottleneck in Italian wolves is still lacking. To fill this gap, we sequenced whole genomes of 13 individuals sampled in the core historical range of the species in Central Italy to conduct population genomic analyses, including a comparison with wolves from two highly-inbred wolf populations (i.e. Scandinavia and Isle Royale). We found that peninsular Italian wolves, despite their recent recovery, still exhibit relatively low genetic diversity, a small effective population size, signatures of inbreeding, and a non-negligible genetic load. Our findings indicate that the peninsular Italian wolf population is still susceptible to bottleneck legacies, which could lead to local inbreeding depression in case of population reduction or fragmentations. This study emphasizes the importance of considering key genetic parameters to design appropriate long-term conservation management plans.

## Graphical Abstract

**Graphical Abstract:**
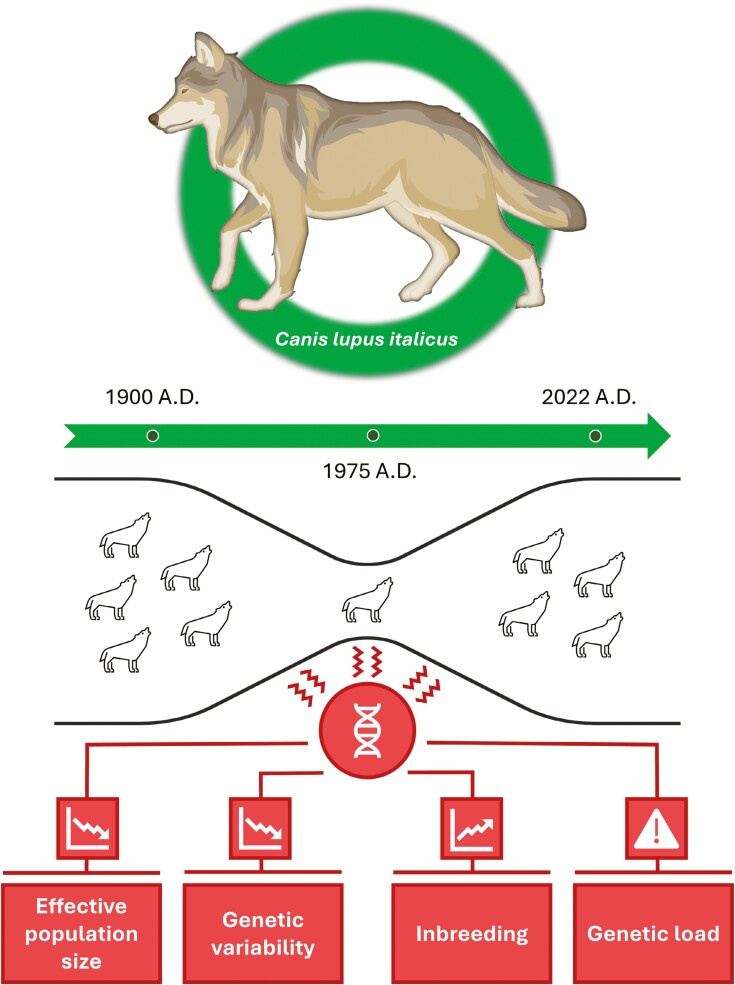

## 1. Introduction

Genetic diversity is one of the three biodiversity pillars (DeWoody et al. 2021), having recently received full recognition at the policy level in the Kunming-Montreal Global Biodiversity Framework (Ma 2023). Preserving genetic diversity and avoiding inbreeding depression is needed to maintain long-term thriving natural populations (Kardos et al. 2021). Therefore, key genetic parameters should be carefully taken into account to evaluate the conservation status of natural populations, especially if they have experienced a severe bottleneck, regardless of any subsequent demographic recovery (DeWoody et al. 2021; Kardos et al. 2021). One key parameter is the effective population size (*N*_e_), which represents an idealized population of randomly mating individuals (Waples 2022) and is linked to genetic diversity loss over time (Charlesworth 2009). Factors deviating from Wright-Fisher model assumptions can cause estimated *N*_e_ significantly different from population size (*N*) (Keller and Reeve 1994; Hedrick and Kalinowski 2000; Charlesworth 2009; Clutton-Brock 2021). Severe bottlenecks may increase genetic drift and inbreeding, potentially impacting population standing genetic variation, and lead to inbreeding depression. Without interpopulation connectivity, new genetic variation can only arise through de novo mutations, which are more likely to quickly drift in isolated small populations, leading to persistently low genetic diversity (Coyne et al. 1997; Wade and Goodnight 1998). Additionally, an increase in homozygosity for deleterious recessive alleles (Charlesworth and Willis 2009) may result in genetic load transitioning from a “masked” to “realized” status, thereby reducing individual fitness with potentially negative effects at the population level (Bertorelle et al. 2022), and thus representing an additional serious conservation challenge in the absence of gene flow (Kardos et al. 2016).

The genetic diversity of wild species has strongly declined in the last century, mainly due to anthropogenic activities that triggered demographic declines and habitat fragmentation (Exposito-Alonso et al. 2022). Due to their life history traits and propensity to generate conflicts with humans, large carnivores are particularly exposed to these negative effects (Boitani and Linnell 2015). Nevertheless, due to recent legal protection, conservation efforts, and improved habitat suitability, large carnivores are currently recovering throughout Europe (Chapron et al. 2014). The wolf (*Canis lupus*), is a paradigmatic case of such a natural recovery (Chapron et al. 2014), due to its ability to disperse rapidly over long distances and anthropogenic landscapes (Ciucci et al. 2009; Boitani and Linnell 2015; Hindrikson et al. 2017). However, while Eastern European wolves have maintained relatively large populations, functionally connected with counterparts in Russia and Asia (Mech and Boitani 2003), populations in Southern Europe, which started to diverge during the Pleistocene, have remained isolated for centuries (Fan et al. 2016).

In particular, prolonged isolation south of the Alps and recurrent bottlenecks from the last glacial maximum until the last century have made the Italian wolf population the most morphologically and genetically differentiated among the European wolf populations (Lucchini et al. 2004; Stronen et al. 2013; Pilot et al. 2014), to be recognized as a distinct subspecies (*C. lupus italicus*) (Randi et al. 2000; Nowak and Federoff 2002; Montana et al. 2017). As expected, genetic drift and inbreeding acting during such prolonged isolation, as well as continuous demographic declines, caused a drastic loss of genetic variability in the population (Pilot et al. 2014). Nevertheless, the last historical bottleneck, which occurred after the Second World War, mainly caused by human persecution, was dramatic from a conservation perspective. The species was brought to the brink of extinction in the late 1960s to 1970s with only a few individuals surviving in the Central-Southern Apennines (Zimen and Boitani 1975). However, due to conservation measures and overall positive attitudes from most of the society, wolves have naturally and rapidly recolonized much of their original range throughout the Italian peninsula over the following five decades (Fabbri et al. 2007), finally reaching the western Alps in the 1990s (Lucchini et al. 2002) and the eastern Alps in the 2010s (Fabbri et al. 2014). Currently, both Alpine and peninsular wolf populations are numerically increasing and are considered as two distinct management units because of their strongly different ecological and socio-economic contexts (Linnell et al. 2007; Marucco et al. 2023; Gervasi et al. 2024): the first is transboundary, and directly connected with the Dinaric-Balkan-Pindus wolf populations, while the second still remains isolated.

However, the peninsular population is still threatened by critical issues such as poaching, illegal killings, and anthropogenic hybridization with the domestic dog, which might affect the gene pool of contemporary wolves (Randi and Lucchini 2002; Verardi et al. 2006; Randi et al. 2014; Pilot et al. 2018; Salvatori et al. 2019, 2020; Harmoinen et al. 2021; Santostasi et al. 2021). The genetic consequences of these population dynamics and threats for the peninsular Italian wolves can be considered well studied through mitochondrial DNA, microsatellite, and high-resolution Single Nucleotide Polymorphism (SNP) panel analyses (Ciucci et al. 2009; Clutton-Brock et al. 2021; Coyne et al. 1997; Danecek et al. 2011; Exposito-Alonso et al. 2022; Hindrikson et al. 2017; Hoban et al. 2024; Hoffmann et al. 2017). Although a few studies have used a single peninsular Italian wolf genome (Fan et al. 2016; Silva et al. 2020), no study has yet provided information at the population level with whole-genome data.

Therefore, to verify if even a recovered wolf population might still reveal bottleneck legacies, that should not be ignored in long-term conservation and management planning, we sequenced good-coverage whole genomes of 13 individuals sampled in the historical stronghold of the peninsular population in Central Italy. We used these newly sequenced genomes to perform, for the first time, comprehensive population genomic analyses, with the aims to: 1) evaluate the current genomic variability and estimate *N*_e_ in the peninsular Italian wolf population; 2) assess trends in the historical *N*_e_; and 3) investigate if inbreeding has significantly affected the peninsular Italian wolf genome-wide variation and its genetic load after the historical bottleneck. Moreover, for comparative purposes, we also included two other highly inbred wolf populations in Scandinavia and Isle Royale (United States). In fact, similarly to Italian wolves, these two wolf populations underwent strong bottlenecks and founder effects (Peterson and Page 1988; Vilà et al. 2003), and currently show whole-genomic signals of deep inbreeding and increased genetic load (Kardos et al. 2017; Robinson et al. 2019; Smeds and Ellegren 2023).

## 2. Materials and methods

### 2.1 Sample collection and DNA extraction

Tissue samples were collected from 13 peninsular Italian wolves between 2007 and 2012 from found-death individuals in the Central Apennines, where historical strongholds of wolves in Italy are located (Zimen and Boitani 1975). The tissue samples were stored in ethanol at −20 °C and subsequently processed in the Conservation Genomics Research Unit at the Fondazione Edmund Mach (FEM). Small fragments of tissue of around 25 mg were extracted using the DNeasy Blood and Tissue Kit (Qiagen) with overnight digestion at 56 °C. The elution was performed at the GLOBE Institute (University of Copenhagen) using two washes of 50 µL of AE buffer, with 10 min of incubation at 37 °C. Until the elution, samples were stored at −20 °C inside the DNeasy Mini spin columns.

### 2.2 Library preparation, amplification, and whole-genome sequencing

Extracts were fragmented in the Covaris LE220 plus Focused-ultrasonicator with the parameters set for 350-bp fragment length. The extracts were diluted to obtain 100 ng concentration and Beijing Genomics Institute (BGI) libraries for Italian wolves were constructed following previously optimized protocols (Mak et al. 2017; Carøe et al. 2018) and using 10 µM adaptors. Libraries were purified using MinElute columns using PE buffer (Qiagen) and eluted in 60 µL of EB buffer. The Polymerase Chain Reaction (PCR) mixture for the peninsular Italian wolf libraries consisted of 20 µL of purified library, 0.2 µM of forward and reverse BGI primers, 2.5 U/µL PfuTurbo Cx HotStart DNA Polymerase, 10 µL of Buffer 10×, 0.08 mg/mL BSA, 0.5 mM of dNTPs (25 µM), and 61.2 µL AccuGene molecular biology water (Lonza, Basel, CH). The amplification of peninsular Italian wolf samples was performed as follows: initial denaturation at 95 °C for 2 min followed by 10 to 12 cycles of 95 °C for 30 s, 60 °C for 30 s, and 72 °C for 110 s, and a final elongation step at 72 °C for 10 min. Peninsular Italian wolf samples were sequenced on one-eighth of a lane each on MGIseq2000 PE150 and DNBSEQ PE150, respectively. Four out of 13 samples have been previously published by Ciucani et al. (2023) (Supplementary Table 1) at a low coverage (ca 3.6×). We resequenced these samples aiming to reach a higher coverage for the purpose of this study.

### 2.3 Dataset

In addition to the 13 peninsular Italian wolves (WIT) genomes sequenced here, we also retrieved genomic data from public databases (NCBI GenBank) (Supplementary Table 1). Therefore, the final dataset compiled for this study comprised 101 modern samples, including 13 WIT (*C. lupus italicus*), 10 Scandinavian wolves (*C. lupus*) (WSC) (Kardos et al. 2017), 11 North American wolves from Isle Royale (*C. lupus*) (WUS) (Robinson et al. 2019), and 67 modern domestic dogs (*C. lupus familiaris*) belonging to 67 different breeds of medium or large size (DOG) (Supplementary Table 1). The 10 Scandinavian wolves were a random subsample of those available (*n* = 96). One African wolf (*Canis lupaster*) and one Golden Jackal (*Canis aureus*) were used as outgroups (OUT) for the genetic load analyses.

### 2.4 Quality control and alignment

We applied a quality control procedure on the sequencing reads, using FastQC (Andrews 2010) to check for possible issues such as low-quality scores and anomalous Guanine-Cytosine (GC) content, and we used multiQC (Ewels et al. 2016) to visualize them. The reads were mapped both onto the wolf reference genome (Gopalakrishnan et al. 2017) and onto the dog reference genome (CanFam3.1 (Lindblad-Toh et al. 2005)), as the “admixture analyses” and the “genetic load analyses” required genomic regions from dogs and outgroups to be also mapped to the dog reference genome. To perform mapping, we set and ran the automated PALEOMIX BAM pipeline (Schubert et al. 2014): first, it indexed each read prefix using SAMtools “faidx” (Danecek et al. 2021); then it removed the specified BGI adapters using AdapterRemoval (Lindgreen 2012); the mapping was done using BWA “mem” algorithm that is suggested for modern samples (Li and Durbin 2009), setting the minimum mapping quality to 0 to retain all the reads in this step; to conclude PCR duplicates were removed using Picard MarkDuplicates (https://broadinstitute.github.io/picard/). After that, we used SAMtools (Danecek et al. 2021) to remove nonprimary alignment reads (samtools view -F 256).

### 2.5 Genotype processing

We used GATK v 4.3.0.0 and referred to GATK Best Practice Workflow to call high-quality genotypes (Van Der Auwera et al. 2013). Then we applied two additional GATK tools for “hard filtering” our genotypes: VariantFiltration (QD < 2.0, FS > 60.0, MQ < 40.0, MQRankSum < −12.5 and ReadPosRankSum < −8.0; settings taken from alternative protocol 2 in GATK Best Practices) to mark the filters, and SelectVariants to apply them. Finally, we applied other filters using VCFtools (Danecek et al. 2011) to keep only biallelic SNPs (flags --remove-indels --max-alleles 2 --min-alleles 2) and to filter for minor allele frequency (MAF), missingness, minimum quality and minimum average depth (flags --maf 0.05 --max-missing 0.9 --minQ 30 --minDP 5 --min-meanDP 5). We used those filters on different datasets according to the assumptions of the downstream analyses (Supplementary Table 2). As “admixture analyses” rely on the assumption that SNPs are not in physical linkage, we performed linkage disequilibrium (LD) pruning using PLINK v 1.90b6.21 (Chang et al. 2015), setting a window size of 10 kb, a step size of 5 bp and an *r*^2^ threshold of 0.5 (flag --indep-pairwise 10 5 0.5). Moreover, to avoid violating the assumptions of random mating when carrying out most population genomic analyses, we used NgsRelate2 to identify and eventually remove closely related individuals (Hanghøj et al. 2019) applying thresholds of KING-robust kinship ≥ 0.20, R0 ≤ 0.1, and R1 ≥ 0.5 (Waples et al. 2019).

### 2.6 Admixture analyses

We explored patterns of genetic differentiation among samples through a preliminary non-model Principal Components Analysis (PCA), calculating PCs with PLINK v 1.90b6.21 (flag --pca). The percentage of variance explained was calculated from the “.eigenval” output, and the first two principal components (PCs) were used for plotting in R v 4.2.1 using “ggplot2” (Wickham 2016). Then, we used ADMIXTURE v1.3.0 which uses a maximum likelihood approach (Alexander et al. 2009) to estimate the proportions of a given number of ancestries (*K*) for each individual. We assumed *K* values from 2 to 6 and set the --cv flag to calculate cross-validation errors (CV) for each *K*. For each *K*, we ran 15 independent iterations with different starting seeds and chose the iterations with the highest likelihood. The best *K* was then chosen based on the lowest CV value among the best iterations. If individuals potentially admixed with dogs were identified, we re-ran the analysis excluding them. Admixed individuals were further investigated within each specific wolf population through a supervised ADMIXTURE analysis (flag --supervised) to confirm their status after reapplying the filters for “admixture analyses”. In case some of these individuals were confirmed as admixed (i.e. hybrids or introgressed with dog), we conducted the downstream analyses by both keeping and removing them to highlight potential differences. As Stefanović et al. (2024) demonstrated pervasive jackal-dog hybridization across the *C. aureus* range, we also checked dog ancestry in the chosen OUT by applying an additional supervised ADMIXTURE analysis.

### 2.7. Genomic variability analyses

To compare the patterns of genomic variation among the three wolf populations, we estimated the observed heterozygosity (Ho) and nucleotide diversity (π). We used ANGSD (Korneliussen et al. 2014) to estimate the heterozygosity of each sample, by calculating the folded site frequency spectrum (SFS) on autosomes. We estimated genotype likelihoods using ANGSD’s GATK (-GL 1) model (doSaf 1), removing bases with a quality score lower than 20 (-minQ 20), and reads with a mapping quality lower than 30 (-minmapq 30). The dog reference genome was used both as a reference and as ancestral (-ref and -anc options). The genome-wide SFS was estimated using the realSFS utility tool provided in ANGSD and subsequently, the final heterozygosity was calculated as the ratio of heterozygous sites/total sites. Since most of the individual genomes did not exhibit a high depth of coverage (>20×), and a recent study has pointed out that low sequencing depth can bias the specific downstream analyses we are interested in (e.g. inbreeding and *N*_e_ estimates; (Kardos and Waples 2024)), we tested the possible correlation between individual heterozygosity and read depth using R v 4.2.1 (Pearson’s correlation test). Subsequently, we tested for possible discrepancies between heterozygosity estimated from genotype likelihood and heterozygosity estimated from variant calling procedures. Nucleotide diversity (π) was estimated on variant sites only, using VCFtools (Danecek et al. 2011). We applied a sliding window approach with a window size of 100 kb (--window-pi 100000), including all the samples for each population.

### 2.8 Demographic analyses

To understand the demographic trend in the three wolf populations over time, we estimated recent historical *N*_e_ in the three wolf populations using the LD linkage disequilibrium method as implemented in GONE (Santiago et al. 2020). GONE exploits the genetic distances among SNPs to estimate *N*_e_ in more recent generations (relying on the information associated with loosely linked SNPs) and historical *N*_e_ (relying on the information associated with tightly linked SNPs), providing reliable estimates of *N*_e_ up to 200 generations ago. In GONE, input parameters were set to their default values, except the average recombination rate that was set to 1.3459 CentiMorgans per Megabase, as obtained from Auton et al. (2013) (CanFam 3.1). We considered generation length in wolves to be 3 to 4 years (Gray et al. 2009; Mech et al. 2016) and ran the analysis on a maximum of 50,000 SNPs per chromosome (maximum value accepted by GONE) with no additional MAF filtering. Analyses in GONE were repeated 20 times to obtain empirical confidence intervals. Although this method may not fully capture the uncertainty introduced by the sampling process (as, for instance, methods based on jackknifing), especially given our reduced sample size, it is able to provide an indication of the uncertainty around our *N*_e_ estimates in GONE (Santiago et al. 2020). Point estimates were summarized using the geometric mean across the values obtained in each replicate. We also used the software currentNe, which is more accurate than GONE for contemporary *N*_e_ (i.e. last 2 to 3 generations) even with small sample sizes (Santiago et al. 2024). CurrentNe produces confidence intervals for *N*_e_ based on artificial neural networks without the need for iterating the analysis. We subsampled the datasets for WIT, WUS, and WSC to 50,000 random SNPs prior to the analyses in current *N*_e_, and used the *N*_e_ estimate obtained only based on LD between chromosomes. To understand how the pedigree of the sampled individuals would affect the *N*_e_ estimation, we carried out the analyses by either including or excluding highly related individuals, under the expectation that in a random sample, the frequency of related individuals is a determinant of the genetic drift signal in the population and therefore, the exclusion of putative relatives from the analyses can upwardly bias the *N*_e_ estimates (Waples and Anderson 2017; Waples 2024).

### 2.9 Inbreeding analyses

We identified runs of homozygosity (ROH), indicative of putative identity-by-descent chromosome segments, in WIT, WSC, and WUS whole-genome data using the window-based approach implemented in PLINK v 1.90b6.21 (--homozyg). We employed a sliding window of 100 kb (--homozyg-kb 100), a threshold that has found favor in population genetics studies of non-model species (Robinson et al. 2019; Hasselgren et al. 2021; Sánchez‐Barreiro et al. 2021). For the remaining parameters, we kept PLINK default values. A minimum of 100 SNPs (--homozyg-snp 100) at a minimum density of 1 SNP per 50 kb was required to call a ROH (--homozyg-density 50). To account for genotyping errors, we allowed up to 1 heterozygous site per 1000 kb window within called ROHs (--homozyg-window-het 1), as per (Ceballos et al. 2018a), and five missing calls per 1,000 kb window within called ROHs (--homozyg-window-missing 5). A length of 1,000 kb between two SNPs was required in order for them to be considered in two different ROHs (--homozyg-gap 1000). We calculated the total length of all ROHs for each individual (SROH) and plotted it on the “x” axis against the number of ROHs for each individual (NROH) on the “y” axis. We estimated the inbreeding coefficient based on ROH (FROH) as the ratio of SROH to the total length of the autosomal genome covered by SNP positions (herein 2,222,501,653 bp) for each individual. We plotted SROH accounting for different ROH sizes (“short”: 100 kb < ROH < 1 Mb; “intermediate”: 1 Mb < ROH < 10 Mb; and “long”: ROH > 10 Mb), to highlight the FROH components. Finally, we calculated ROHs coalescence time aiming to define the time at which the ROHs were likely formed. To do so, we used the formula *L* = 100/2*t* cM (Thompson 2013) where *L* is the length of the ROH, cM is the recombination rate, and *t* is the time of coalescence in generations. If demographic analyses revealed some bottleneck, we checked the impact of the ROHs that have been formed during and immediately after the bottleneck (five generations later) on the population SROH.

### 2.10 Genetic load analyses

We used publicly available short-read data from two OUT, *C. lupaster* (African wolf, SRA accession: SAMN10199001) and *C. aureus* (Golden Jackal, SRA accession: SAMN10180427) to polarize SNPs, defining the “ancestral” and ‘derived’ states for each variant. To do so, we used est-sfs (Keightley and Jackson 2018), a tool implementing a maximum likelihood method to infer the unfolded site frequency spectrum (the uSFS) and the ancestral state probabilities for our OUT data. Using a custom script, we obtained the est-sfs input file and extracted the ancestral state with the highest probability for each variant from the output file. If two bases had the same probability of being ancestral, the script randomly picked one of the two. We used the domestic dog genome annotation gtf, cDS and protein files (CanFam3.1; Ensembl release 104 https://may2021.archive.ensembl.org/Canis_lupus_familiaris/Info/Index) to build custom snpEff v.4.3.183 (Cingolani et al. 2012) and SIFT4G v.6.084 (Vaser et al. 2016) databases with default settings. We then annotated and predicted the effects of variants using the aforementioned tools. Following annotation, we retained variants where the derived state matched the alternative allele in the dog reference genome. This ensured that the derived alleles had the deleterious effects indicated for the alternative alleles of the dog reference genome. We classified putatively deleterious variants into three categories: 1) Low-impact (LOW) variants likely to be not deleterious (i.e. synonymous), 2) Moderate-impact (MODERATE) variants likely to modify protein effectiveness (i.e. nonsynonymous), and 3) High-impact (HIGH) variants likely to disrupt protein function (i.e. loss of function LoF, stop codons, splice donor variant and splice acceptor, or start codon lost) (Linnell et al. 2007). We used SIFT (Sorting Intolerant From Tolerant) score to discriminate MODERATE in tolerated nonsynonymous (SIFT score ≥ 0.05) (MOD-TOL) and putatively deleterious nonsynonymous (SIFT score < 0.05) (MOD-DEL) variants. Then we used a custom script to estimate individual allele frequencies and genotype counts of LOW, MOD-TOL, MOD-DEL, and HIGH-impact variant-derived alleles, for each wolf in the dataset. In particular, the count of heterozygous genotypes represented the “masked” load, which quantifies the potential loss of fitness due to (partially) recessive deleterious mutations that may become expressed in future generations. The count of homozygous genotypes for the derived alleles represented the “realized” load, which reduces fitness in the current generation; the sum of “masked” and “realized” load represented the “total” load (Bertorelle et al. 2022).

## 3. Results

**Fig. 1.**
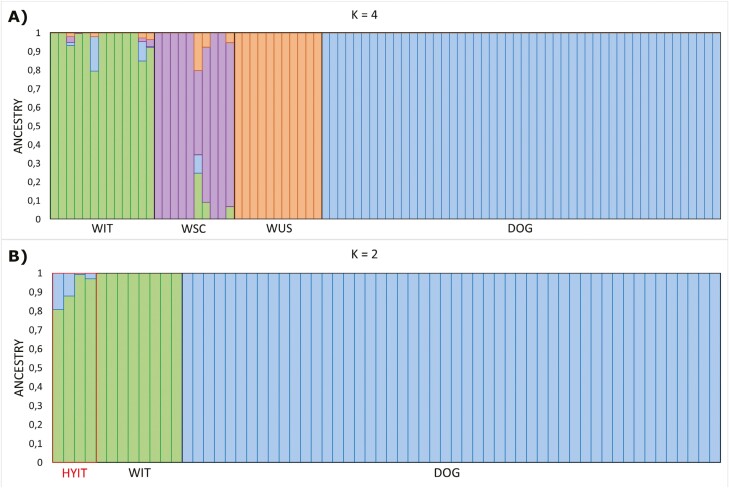
ADMIXTURE clustering analyses showing: A) unsupervised ADMIXTURE results for modern dogs and each studied wolf population, and potential admixed individuals, assuming four ancestry components (*K* = 4); B) supervised ADMIXTURE results conducted to validate potentially admixed WIT (HYIT = Italian wolf-dog hybrid) using non-admixed WIT individuals, and non-admixed DOG individuals as populations with known ancestry. Vertical bars correspond to different samples, and the colors represent the estimated ancestry components and proportions.

We sequenced 13 peninsular Italian wolves (WIT) at an average of 15× coverage and recovered 6,636,110 SNPs. Overall, the dataset we used for the analyses ranged from 555,575 to 8,518,995 SNPs, depending on the analysis and the specific populations included in the analyses (Supplementary Table 2). We did not find any highly related pairs within the sampled population. However, we opted to exclude one individual who displayed two out of three relatedness indexes beyond the threshold along with a reduced genotype quality (Supplementary Fig. 1). We first explored population structure and admixture, aiming to verify the identity of the samples belonging to Italian (WIT), Scandinavian (WSC), and Isle Royale (United States; WUS) populations, and to evaluate if there was dog ancestry in their genomes. The first two components of the explorative PCA explained 54.76% of the genetic variability in the dataset, demonstrating a clear separation between wolves and dogs along the first PC (PC1—41.15%). The second PC (PC2—13.61%) revealed the distinct clustering among the three wolf populations (Supplementary Fig. 2). The ADMIXTURE clustering mirrored the PCA results. We performed CV for the admixture analyses (*K* = {2…7}) and found that our dataset has the lowest CV error when estimating four ancestry clusters (Supplementary Fig. 3). With four ancestry clusters, each wolf population formed a discrete cluster, distinct from the dog cluster. Admixture analysis also revealed 4 WIT, 1 WSC, and 17 DOG individuals as potentially admixed. Re-running the software without the 17 admixed DOG individuals returned the same admixed wolves (Fig. 1A). A “supervised” ADMIXTURE approach confirmed that two of the four admixed WIT had more than 10% DOG ancestry, while the other two shared a smaller (1% to 3%) DOG ancestry proportions (Fig. 1B). The only admixed WSC individual had been previously identified as an immigrant wolf (Kardos et al. 2017), suggesting that the dog ancestry proportion can be explained with wolf ancestry not represented in our reference dataset. Both *C. lupaster* and *C. aureus* genomes (OUT) did not show dog ancestry (Supplementary Fig. 4), and for this reason, they have been confirmed as valuable outgroups for genetic load analyses. Nevertheless, even though we ran the downstream analyses excluding the admixed individuals, either keeping or removing admixed individuals did not significantly affect the results.

### 3.1 Genome-wide patterns of variation

We estimated the genome-wide variation for the WIT in comparison with the other highly inbred wolf populations. Initially, we validated heterozygosity estimation by demonstrating the absence of significant correlation with read depth (*P* value = 0.19) (Supplementary Fig 5). Furthermore, when comparing heterozygosity estimates derived from the genotype likelihoods and those from the called genotypes across the three populations, a consistent pattern emerged. WSC had the lowest observed heterozygosity (Ho = 0.00149 ± 0.00025) estimate, followed by WIT and WUS with Ho = 0.00152 (±0.00018) and Ho = 0.00178 (±0.00019), respectively, (Fig. 3). Nucleotide diversity estimate was lowest in WIT (π = 0.00082 ± 0.0011) and highest in WSC (π = 0.00108 ± 0.00038), with WUS of intermediate value (π = 0.00102 ± 0.00043) (Supplementary Fig. 6). Applying the Wilcoxon signed-ranked test, we did not find any difference in Ho and π in the three testes wolf populations.

### 3.2 Recent historical and contemporary *N*_e_ estimates

**Fig. 2.**
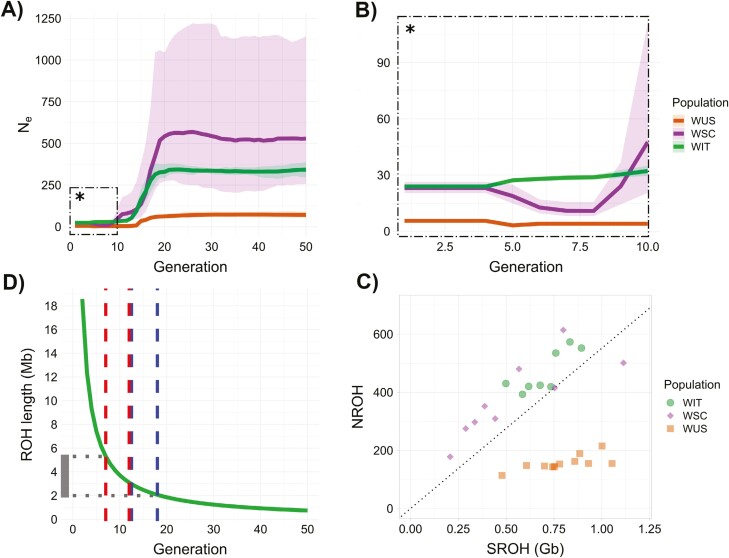
A) *N*_e_ estimates across 20 replicated datasets up to 50 generations ago, each one representing a random sample of approximately 50,000 SNPs per chromosome. Lines represent geometric mean values across replicates, shades are 95% confidence intervals. B) *N*_e_ estimates zoomed for the past 10 generations. C) Number of ROH (NROH) (“y” axis) compared to the sum of the length of ROH (SROH) (“x” axis) across the autosomes. Each individual is represented by a colored shape, while meanings of colors and shapes are specified in the legend on the right. The dotted line represents the intercept, with a slope determined by the ratio of the difference in the range of the NROH variable to the difference in the range of the SROH variable. D) ROH coalescence time up to 50 generations ago in the peninsular Italian wolf population WIT, expressed as a function of ROH length (“y” axis) and generations before present (“x” axis). The right window represents the generation window when the bottleneck occurred, while the left window represents the five generations immediately after the bottleneck. The dotted line and bar on the "y" axis represent the ROH size generated during the aforementioned generation window. 27.14% of SROH belongs to ROHs generated during this time frame.

The demographic analyses carried out with GONE showed that WUS had the smallest estimated *N*_e_, with *N*_e_ below 10 during the most recent generations, and *N*_e_ 62 to 71 from 20 to 50 generations before the present. WIT and WSC showed similar *N*_e_ values (24 to 32 for WIT, 23 to 50 for WSC) for the last 10 generations, with a severe bottleneck occurring between 12 and 18 generations for WIT and 10 to 20 for WSC. From 20 to 50 generations before the present, both populations showed a constant and stable trend, but significantly different *N*_e_ estimates: *N*_e_ in WIT ranged between 330 and 344, and in WSC ranged between 508 and 584, although with much statistical uncertainty, as shown in Fig. 2A and B. Contemporary *N*_e_ estimates (i.e. referring to the last 2 to 3 generations) obtained with currentNe were equal to 10.4 (90% CI: 7.6 to 14.3) for WIT, 14.4 (90% CI: 9.3 to 22.2) for WSC, and 7.4 (90% CI: 5.5 to 10.0) for WUS (Supplementary Table 3). The exclusion of the highly-related WIT individual from the analyses did not significantly affect recent historical *N*_e_ estimates (i.e. last 10 generations: 29 to 48; last 20 to 50 generations: 334 to 386) and contemporary *N*_e_ estimates (11.89; 90% CI: 8.4 to 16.9).

### 3.3 Runs of homozygosity

**Fig. 3.**
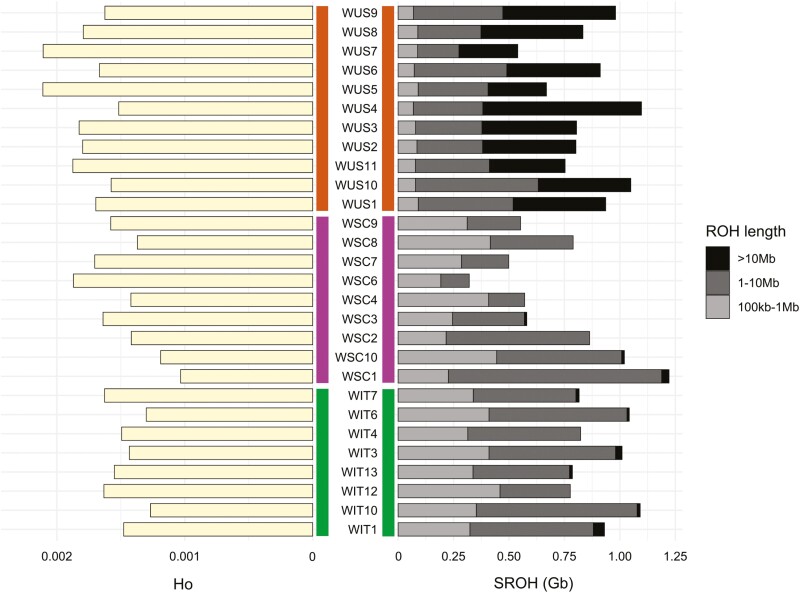
Barplot representing observed heterozygosity estimates (Ho), on the left, and SROH estimates, on the right, for peninsular Italian wolves (WIT), Scandinavian wolves (WSC), and North American wolves from Isle Royale (WUS). The SROH of different ROH sizes is highlighted with a gray-to-black palette.

WIT had both the highest number of estimated ROHs for each individual (NROH) (mean = 1,374.50) and total length of estimated ROHs for each individual (SROH) (mean = 909.47 Mb), followed by WSC (mean NROH = 1,125.88; mean SROH = 713.03 Mb), and WUS (mean NROH = 420.64; mean SROH = 852.33 Mb). We chose the SROH vs. NROH plot based on ROH > 500 kb, given it better represents the demographic history of these samples, after checking different options (ROH > 100 kb, ROH > 1 Mb, ROH > 2 Mb) (Fig. 2C). Individual genomic inbreeding coefficients (FROH) were plotted accounting for all the estimated ROH (>100 kb) (Supplementary Fig. 7). WIT had the highest estimated FROH (mean = 0.409), followed by WUS (mean = 0.380), and WSC (mean = 0.321). When plotting SROH accounting for different ROH sizes (Fig. 3), WIT had the highest estimated SROH for “short” ROHs (mean = 355.43 Mb), followed by WSC (mean = 305.43 Mb) and WUS (mean = 81.12 Mb) (WIT-WUS two-tailed Mann–Whitney *U* [MWU] test *P* value = 1.191e−05; WSC-WUS MWU test *P* value = 2.646e−05). Upon considering intermediate size regions, WIT showed the highest estimated SROH (mean = 502.48 Mb), followed by WSC (mean = 401.45 Mb), and WUS (mean = 348.17) (WIT-WUS MWU test *P* value = 0.001773). When accounting for long ROHs, WUS clearly separated with a higher estimated SROH (mean = 423.04 Mb) compared to WIT (mean = 11.88 Mb) and WSC (mean = 6.13 Mb) (WUS-WIT MWU test *P* value = 0.0001928; WUS-WSC MWU test *P* value = 0.0001615). The coalescence time calculation for ROHs revealed that 100 kb ROHs likely formed more than 350 generations ago, 1 Mb ROHs likely formed 37 generations ago, and 10 Mb ROHs likely formed four generations ago (Supplementary Fig. 8). During and immediately after the bottleneck, WSC formed ROHs of 1.7 to 7.4 Mb that contributed to 32.31% of the population SROH; while WIT formed ROHs of 2 to 5.3 Mb that contributed to 27.14% of the population SROH (Fig. 2D).

### 3.4 Genetic load

**Fig. 4.**
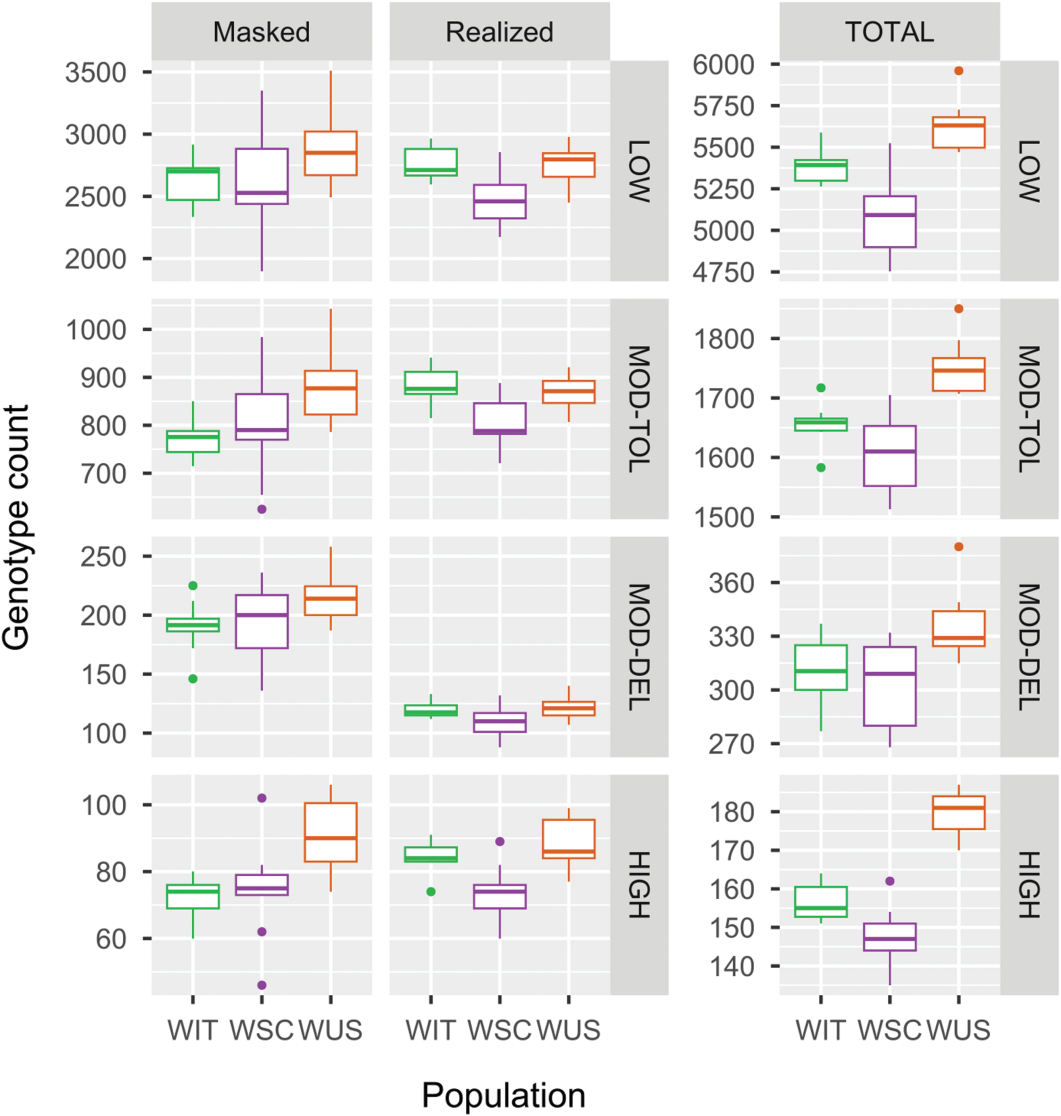
Boxplots representing genetic load estimates based on genotype counts (“x” axis) of heterozygotes (“masked load”) and homozygous genotypes for deleterious alleles (“realized load”) for different degrees impact variants (LOW = low-impact variants, likely not deleterious; MOD-TOL = moderate but tolerated impact variants, likely not deleterious; MOD-DEL = moderately deleterious impact variants, likely to modify protein effectiveness; HIGH = high-impact variants, likely to disrupt protein function) in each wolf population (“y” axis). The “total load” represents the sum of “masked” and “realized” load.

Analysis of derived allele frequencies among individuals (Supplementary Fig. 9) revealed a notable prevalence of likely-not-deleterious alleles (LOW and MOD-TOL) compared to likely deleterious alleles (MOD-DEL and HIGH) across all three populations. Particularly, the most deleterious alleles (HIGH) were found to be the least common. Additionally, genotype count estimations demonstrated a consistent trend across all mutation types, except for WIT, which exhibited the highest average counts for both “masked” LOW and “realized” MOD-TOL alleles (Fig. 4). When focusing on deleterious mutations with significant potential impact on individual fitness (MOD-DEL and HIGH), WUS exhibited the highest load, both “masked” (HIGH: WUS-WIT MWU test *P* value = 0.001262; WUS-WSC MWU test *P* value = 0.01086) and “realized” (HIGH: WUS-WSC MWU test *P* value = 0.004225). Following WUS, WIT showed a similar estimate of “realized” load for both MOD-DEL and HIGH variants (WIT-WSC MWU test *P* value = 0.01373), while WSC demonstrated a comparable estimate of “masked” load for MOD-DEL and HIGH variants. The “total” genetic load assessment positioned WIT as intermediate between WSC and WUS for both MOD-DEL (WIT-WUS MWU test *P* value = 0.0132; WSC-WUS MWU test, *P* value = 0.01089) and HIGH-impact variants (WIT-WSC MWU test *P* value = 0.01589; WIT-WUS MWU test *P* value = 0.0003185; WSC-WUS MWU test, *P* value = 0.0001906).

## 4. Discussion

Population size (*N*) is a functional parameter to assess numerical population trends (Hoffmann et al. 2017). However, to better investigate the conservation status of a population it is necessary to also evaluate genetic and genomic factors, and how they might have been affected by demographic dynamics (DeWoody et al. 2021; Kardos et al. 2021). For example, severe bottlenecks can strongly reduce effective population size (*N*_e_) while facilitating inbreeding. This can determine increased homozygosity for deleterious alleles, which might be phenotypically expressed and affect individual fitness, therefore potentially leading to inbreeding depression and reducing long-term local adaptive potentials. It has been amply demonstrated that the signatures of genomic erosion associated with past bottlenecks can persist even in populations experiencing a geographic re-expansion and a demographic recovery (Abascal et al. 2016; Abadía-Cardoso et al. 2017; Ochoa et al. 2022; Adams and Edmands 2023). Therefore, in this research, we focused on the emblematic case study of the peninsular Italian wolf population (WIT), by applying whole-genome approaches to investigate bottleneck legacies on its current genetic diversity.

Comparative genomic variability analyses with other two highly-inbred wolf populations (Scandinavian—WSC; Isle Royale, United States—WUS) known to be affected by inbreeding depression, showed that peninsular Italian wolves must be placed among the most genetically eroded wolf populations worldwide. These findings confirm previous studies obtained using traditional genetic markers (Pilot et al. 2014; Jan et al. 2023), genome-wide SNP panels (Stronen et al. 2022), or a single whole genome (Fan et al. 2016).

The recent historical *N*_e_ we estimated for WIT showed a significant reduction during 12 to 18 generations before sampling, that, assuming a generation time of 3 to 4 years (Gray et al. 2009; Mech et al. 2016), occurred between 1938 and 1974. These findings align well with known historical demographic trends of Italian wolves (Cagnolaro 1974; Zimen and Boitani 1975), indicating that they disappeared from Northern Italy and Sicily immediately after World War II (Cagnolaro 1974), afterward reaching the minimum population size of about only 100 individuals, in small mountain islets along the Central and Southern Apennines (Zimen and Boitani 1975). Our recent historical and contemporary *N*_e_ estimates in WIT do not parallel current *N* estimates (Galaverni et al. 2016; Gervasi et al. 2024) (Fig. 2). This discrepancy might be due to a persisting bottleneck signal in the sampled individuals, suggesting enduring low viability and limited adaptive potential despite the demographic recovery of the population. We cannot exclude, however, that the localized sampling area, compared to the entire Italian peninsula, and the species life history traits, characterized by few breeders and high juvenile mortality, may contribute to the low *N*_e_ estimate and the high *N* estimate. Additionally, a slight downward bias in the *N*_e_ estimates may partly be expected due to the inclusion of individuals sampled at different times (2007 to 2012), which violates the assumption of discrete generations associated with all *N*_e_ estimation methods (Nunney 1993; Waples 2016; Kardos and Waples 2024).

The ROH analyses showed that bottleneck and inbreeding deeply affected the genomic makeup of the peninsular Italian wolf population, corroborating previous findings based on a single genome (Fan et al. 2016). However, our study also provides a robust population-level perspective over time, confirming that WIT represents one of the most inbred populations both at the Eurasian scale (Salado et al. 2023) and at the global scale (Fan et al. 2016; Robinson et al. 2019). The SROH vs. NROH analyses confirmed a general insight into WIT demographic history. Individual distribution compared to the intercept line (Ceballos et al. 2018b) suggested that bottleneck and inbreeding affected the population. The SROH composition, in terms of ROH length, and ROHs coalescence time could provide us with information about inbreeding and its magnitude (Ceballos et al. 2018b). WIT exhibited the highest SROH estimate for both the shortest ROHs (100 kb), which are less likely to significantly impact individual fitness compared to more recent autozygous segments (Stoffel et al. 2021), and also for intermediate ROHs (1 to 10 Mb long), formed between 4 and 37 generations ago and suggesting a strong bottleneck and post-bottleneck inbreeding.

Our genetic load analyses showed that WIT has a slightly lower realized load compared to deeply-inbred WUS, for both highly (HIGH) and moderately deleterious (MOD-DEL) impact variants, indicating that negative impacts are still acting on individual fitness. This result suggests that purifying selection hasn’t strongly worked, and gene flow couldn’t act as well, given the ongoing isolation of the peninsular Italian population. In fact, while genetic connectivity between the Italian wolves and wolves from the Dinaric-Balkan-Pindus population has been only recently reinstated in the eastern Alps (Marucco et al. 2023), no such connectivity has been documented in peninsular Italy (Linnell et al. 2007; Marucco et al. 2023). The post-bottleneck masked load represents the residual load accumulated by the ancestral population and it may be proportional to the historical *N*_e_ (Van Oosterhout 2020; Bertorelle et al. 2022). For this reason, we expected WIT to show the lowest masked load values, since WSC had a recent historical *N*_e_ estimate higher than WIT, while WUS, originating from the Great Lakes and Ontario wolf population exhibited a higher historical *N*_e_ estimate than WIT in a previous study (vonHoldt et al. 2023). Also in this context, the composition of WSC and WUS (i.e. each population including a few individuals sampled over 25 years) could bias *N*_e_ estimation (mostly likely downward) (Kardos and Waples 2024), but such potential bias should not prevent us from observing general demographic patterns. Considering that WSC masked load estimates could be inflated by immigrants’ genomic variation as well (Smeds and Ellegren 2023), the lack of difference between them and WIT suggests that the latter should not be underestimated. For these reasons, the current WIT genetic load could represent a previously unaccounted but potentially serious conservation issue in case of local fragmentation and reduction. Further genomic investigation, however, should include pre-bottleneck WIT samples to have a proper comparison of genomic variation through time.

Finally, our genomic admixture analyses showed 4 out of 13 of the randomly collected WIT individuals exhibiting >1% of dog ancestry, with two of them exceeding 10%. These findings are consistent with the hypothesis that pervasive wolf-dog hybridization in the peninsular Italian wolf population might have occurred during the recent wolf population expansion when wolf encounters and interbreeding with dogs might have been more likely (Galaverni et al. 2017). Although wolf-dog hybridization has been already documented in Italy using genetic and genome-wide markers (Randi and Lucchini 2002; Verardi et al. 2006; Caniglia et al. 2013; Randi et al. 2014; Galaverni et al. 2017; Pilot et al. 2018; Salvatori et al. 2019, 2020; Harmoinen et al. 2021; Ciucani et al. 2023), our study provides the first evidence based on complete whole genomes. Despite excluding admixed individuals did not significantly affect the results obtained in this study, a larger dataset of whole-genome data will be fundamental to disentangle the effects of this phenomenon on the standing genetic variation. Additionally, further research should explore the evolutionary and ecological implications that the introgression of domestic genes might have on the long-term survival of such a morphologically and genetically unique wolf population.

Our study provides insights into the peninsular Italian wolf population through comprehensive population genomics analyses. Although the individuals we typed were sampled in the core of the historical range in the central Apennines, further studies should enlarge the geographic scope of sampling throughout the whole subspecies distribution in the peninsula. Nevertheless, it has to be considered that unrelated individuals might belong to different packs and regions, and no substructure has been revealed in the peninsular Italian wolf population (Fabbri et al. 2014). For these reasons, we can consider our samples as representative of the peninsular Italian wolf population.

Despite demographic growth and range expansions, wild populations could still be susceptible to eventual geographic fragmentations, numerical reductions, and natural or human-induced environmental changes. Our findings suggest that, although inbreeding depression has not been directly documented in the peninsular Italian wolf population, the reduced genetic diversity, significant inbreeding signatures, and the non-negligible genetic load that we disclosed could lead to fitness decreasing in case of other future demographic contractions or geographic fragmentations. Moreover, given the difficulties in detecting inbreeding depression in natural populations, we cannot exclude that it may already be occurring to some extent at the local scale. In this context, we maintain that, from a conservation-wise perspective, the protection level of wolves should not be defined only considering demographic data but especially their genomic makeup. Including information about genetic variability, effective population size, inbreeding, genetic load, and hybridization rates among criteria to assess population conservation status would be relevant even to design specific management measures from a wide to a local scale (Brüniche-Olsen et al. 2018; Garner et al. 2020; Thurfjell et al. 2022; Hoban et al. 2024). Accordingly, we also maintain that preserving genetic diversity, after receiving full recognition in the Kunming-Montreal Global Biodiversity Framework (Ma 2023), should be more explicitly and specifically stated among the conservation goals in relevant international (i.e. Bern Convention), European (i.e. Habitats Directive) and national conservation treaties and laws.

## Supplementary material

Supplementary material is available at *Journal of Heredity* online.

esae041_suppl_Supplementary_Data

## Data Availability

All the data and codes processed and produced in this work have been submitted to Dryad (DOI: 10.5061/dryad.ngf1vhj2f; https://datadryad.org/stash/share/Ql2eeqaemPKckr_5jYxocEvAPAekaVrWMO7hZy3rlgA). Raw DNA sequence reads of whole genomes generated in this study have been submitted to European Nucleotide Archive (ENA) (Accession: PRJEB80121; Secondary Accession: ERP164161).
