# Supplementary material for: Beyond population size: Whole-genome data reveal bottleneck legacies in the peninsular Italian wolf
Source: J Hered. 2024 Aug 27;116(1):10–23. doi: 10.1093/jhered/esae041 (PMC11700593; doi:10.1093/jhered/esae041)
Supplement: esae041_suppl_Supplementary_Data [file esae041_suppl_supplementary_data.zip › esae041_suppl_Supplementary_Figures_1/Supplementary_list.docx]

**SUPPLEMENTARY MATERIAL**

**Beyond population size: whole-genome data reveal bottleneck legacies in the peninsular Italian wolf**

Daniele Battilani [1,3,4], Roberta Gargiulo [2], Romolo Caniglia [3], Elena Fabbri [3], Jazmin Ramos Madrigal [4], Claudia Fontsere [4], Marta Maria Ciucani [4], Shyam Gopalakrishnan [4], Matteo Girardi [5], Ilaria Fracasso [5], Matteo Mastroiaco [1], Paolo Ciucci [1], Cristiano Vernesi [5]

**List of supplementary material:**

*Supplementary Fig. 1 –* Pairwise comparisons to identify highly-related individuals. The plots display two combinations of three kinship statistics used in Waples et al. (2019) method to avoid ascertainment bias: R1 vs R0 and R1 vs KING-robust. The couple of individuals exhibiting R0 & KING-robust over has been highlighted in red, while the removed individual due to reduced genotype quality was WIT5.

*Supplementary Fig. 2* – PCA representing genetic structure between the populations under analyses. PC1 (41.15% explained variation) demonstrates a clear separation between wolves and dogs. PC2 (13.61% explained variation) confirmed distinct clustering among the three wolf populations.

*Supplementary Fig. 3* – Cross-validation error (CV) of the best ADMIXTURE iterations for each K (number of assumed clusters).

*Supplementary Fig. 4* – supervised ADMIXTURE checking for dog ancestry in the selected outgroups (OUT). Both *Canis aureus* (SAMN03366713) and *Canis lupaster* (SAMN10199001) did not exhibited domestic ancestry.

*Supplementary Fig. 5* – Scatterplot with individual heterozygosity as a function of read depth (Coverage), aimed to validate genotype calling procedures. The Pearson’s correlation test confirmed the absence of significant correlation between the two variables (p-value = 0.19).

*Supplementary Fig. 6* – Nucleotide diversity (π) variation along the genome of each population under study, given by the synthesis of each individual result. For each wolf population, the reported mean represents π averaged across all the windows summed together.

*Supplementary Fig. 7* – Fraction of runs of homozygosity on total genome length (FROH) estimated accounting for ROH>100 kb in each wolf population.

*Supplementary Fig. 8* – Estimated coalescence time of all kinds of ROH (short, intermediate and long) from the number of generations associated with minimum size ROH (100 kb) to present time. Coalescence time has been estimated used the formula L = 100/2t cM (Thompson 2013) where L is the length of the ROH, cM is the recombination rate and t is the unknown time of coalescence in generations.

*Supplementary Fig. 9* – Low (LOW), moderate but tolerated (MOD-TEL), moderate but deleterious (MOD-DEL), and high (HIGH) impact variants allele frequencies in each wolf population individuals.

*Supplementary Table 1* – S1. Italian wolves (WIT) metadata.

*Supplementary Table 1* – S2. Scandinavian wolves (WSC) metadata.

*Supplementary Table 1* – S3. Isle Royale wolves (WUS) metadata.

*Supplementary Table 1* – S4. Domestic dogs (DOG) metadata.

*Supplementary Table 1* – S5. Outgroups (OUT) metadata.

*Supplementary Table 2* - Genotype filtering summary for each kind of analyses.

*Supplementary Table 3* - Contemporary (2-3 generations before sampling) *N_e_* estimates in the Italian wolf (WIT), Scandinavian wolf (WSC), and Isle Royale wolf (United States; WUS) populations, using currentNe. Values inside brackets represent 90% confidence intervals (CIs).
