# Supplementary material for: Beyond population size: Whole-genome data reveal bottleneck legacies in the peninsular Italian wolf
Source: J Hered. 2024 Aug 27;116(1):10–23. doi: 10.1093/jhered/esae041 (PMC11700593; doi:10.1093/jhered/esae041)
Supplement: esae041_suppl_Supplementary_Data [file esae041_suppl_supplementary_data.zip › esae041_suppl_Supplementary_Figures_1/Supplementary_Table3_currentNe.docx]

| **Population** | **Contemporary *N_e_* (± 90% CIs)** |
| --- | --- |
| **WIT** | 10.4 (7.6-14.3) |
| **WSC** | 14.4 (9.3-22.2) |
| **WUS** | 7.4 (5.5-10.0) |

*Table 3* – Contemporary (2-3 generations before sampling) *N_e_* estimates in the Italian wolf (WIT), Scandinavian wolf (WSC), and Isle Royale wolf (United States; WUS) populations, using currentNe. Values inside brackets represent 90% confidence intervals (CIs).
