# Supplementary figures and images for: Beyond population size: Whole-genome data reveal bottleneck legacies in the peninsular Italian wolf
Source: J Hered. 2024 Aug 27;116(1):10–23. doi: 10.1093/jhered/esae041 (PMC11700593; doi:10.1093/jhered/esae041)

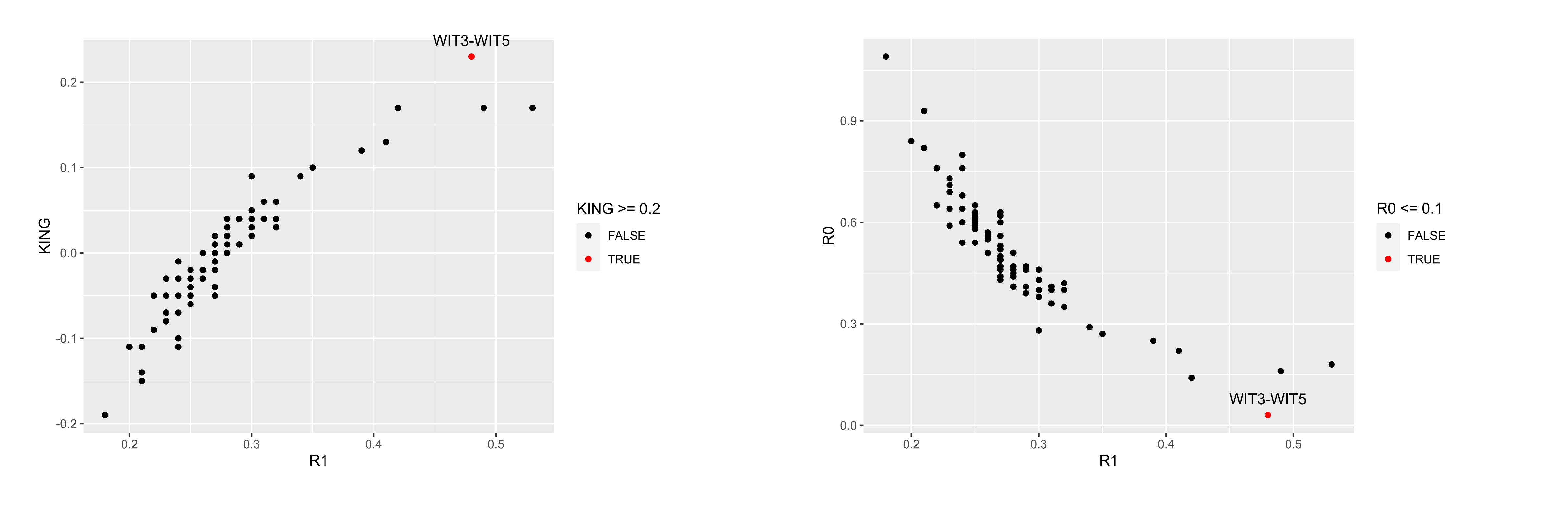

Supplement: esae041_suppl_Supplementary_Data [file esae041_suppl_supplementary_data.zip › esae041_suppl_Supplementary_Figures_1/Supplementary_Fig1.jpeg]

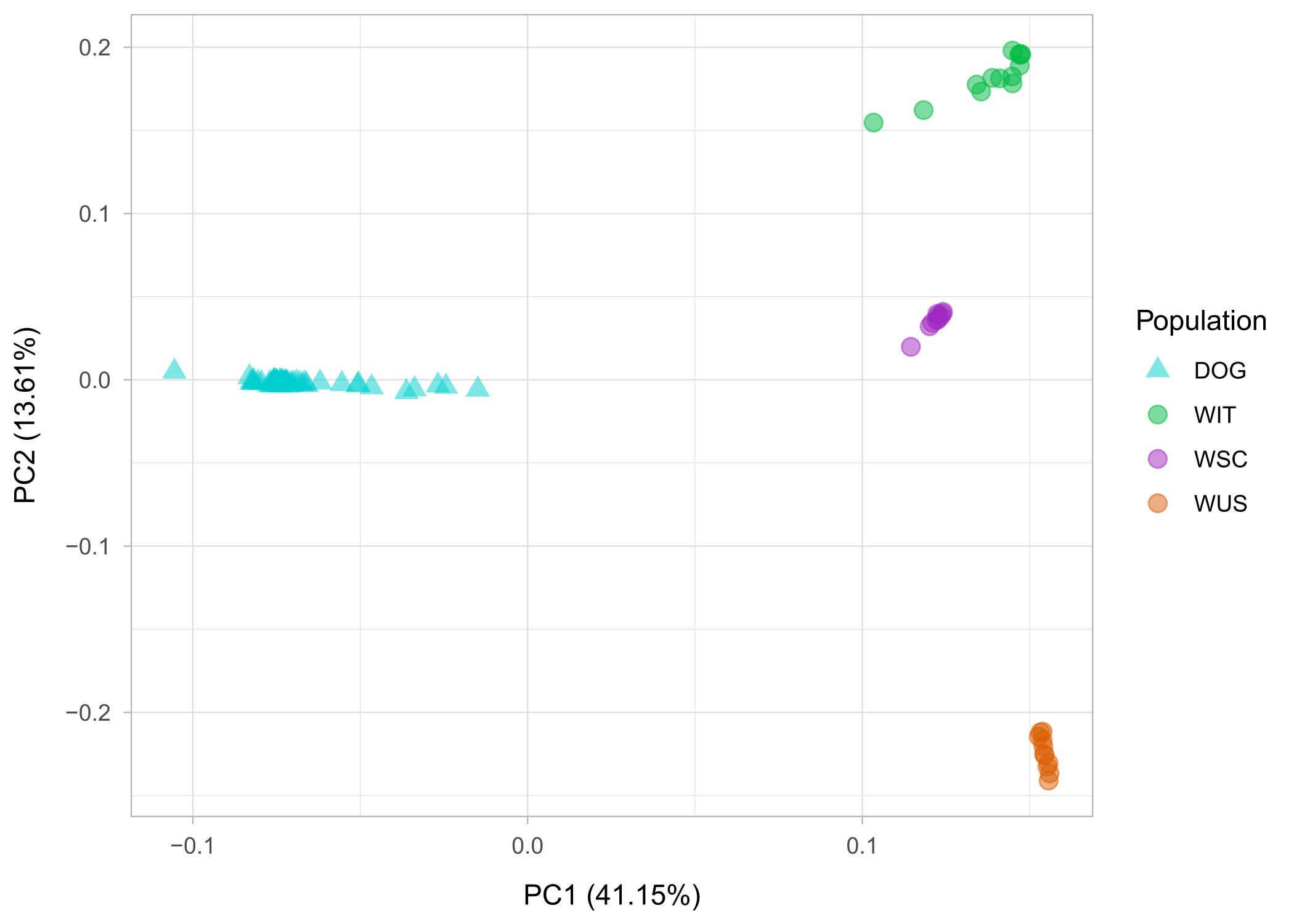

Supplement: esae041_suppl_Supplementary_Data [file esae041_suppl_supplementary_data.zip › esae041_suppl_Supplementary_Figures_1/Supplementary_Fig2.jpeg]

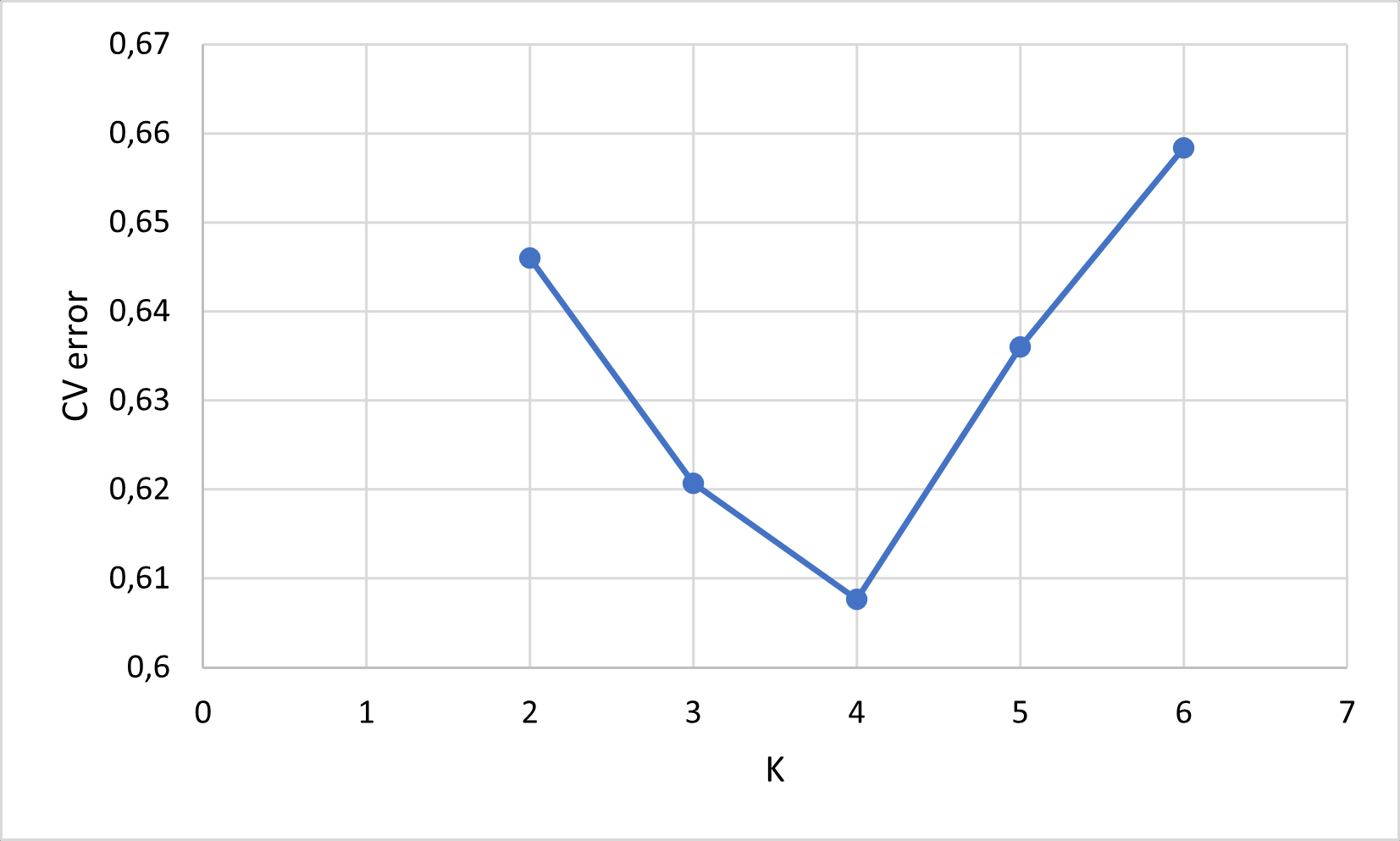

Supplement: esae041_suppl_Supplementary_Data [file esae041_suppl_supplementary_data.zip › esae041_suppl_Supplementary_Figures_1/Supplementary_Fig3.jpeg]

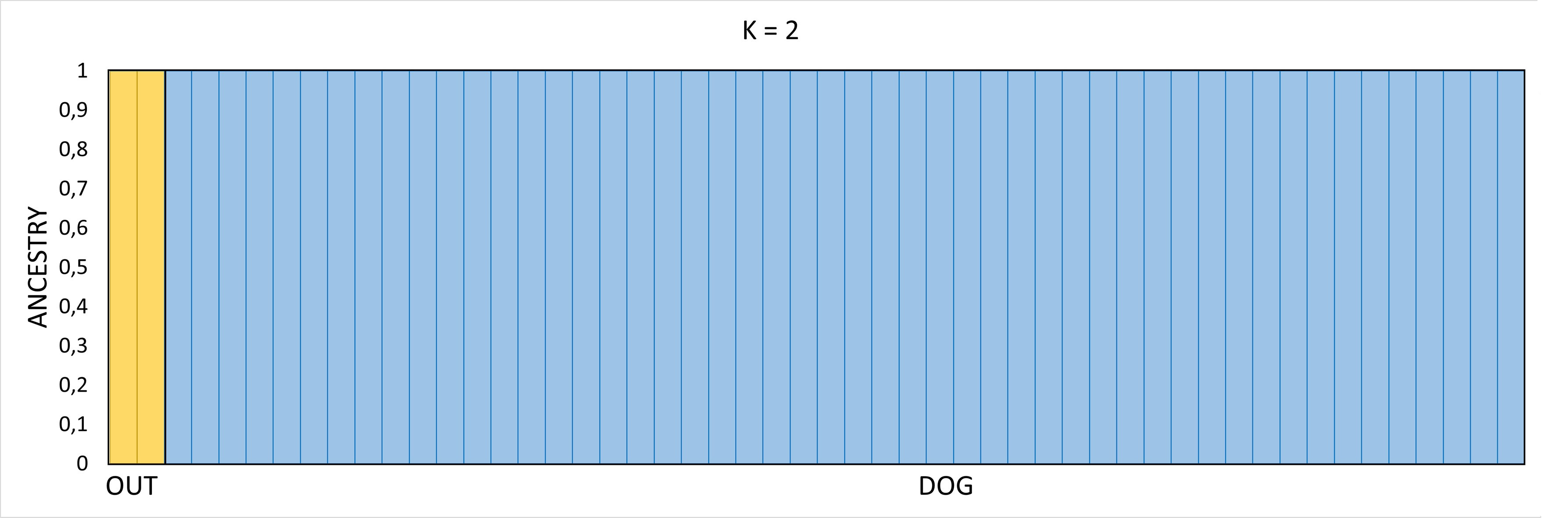

Supplement: esae041_suppl_Supplementary_Data [file esae041_suppl_supplementary_data.zip › esae041_suppl_Supplementary_Figures_1/Supplementary_Fig4.jpg]

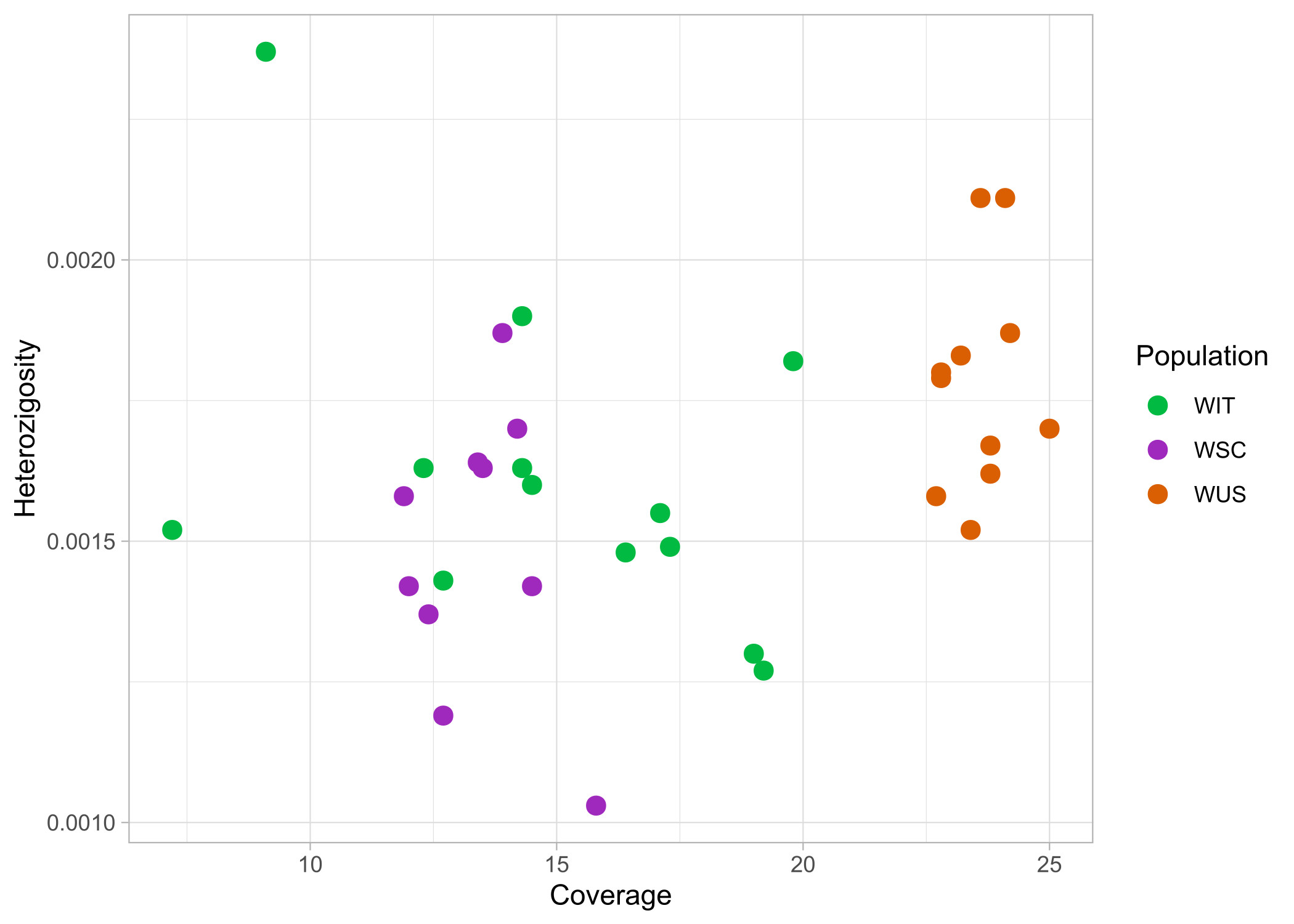

Supplement: esae041_suppl_Supplementary_Data [file esae041_suppl_supplementary_data.zip › esae041_suppl_Supplementary_Figures_1/Supplementary_Fig5.jpeg]

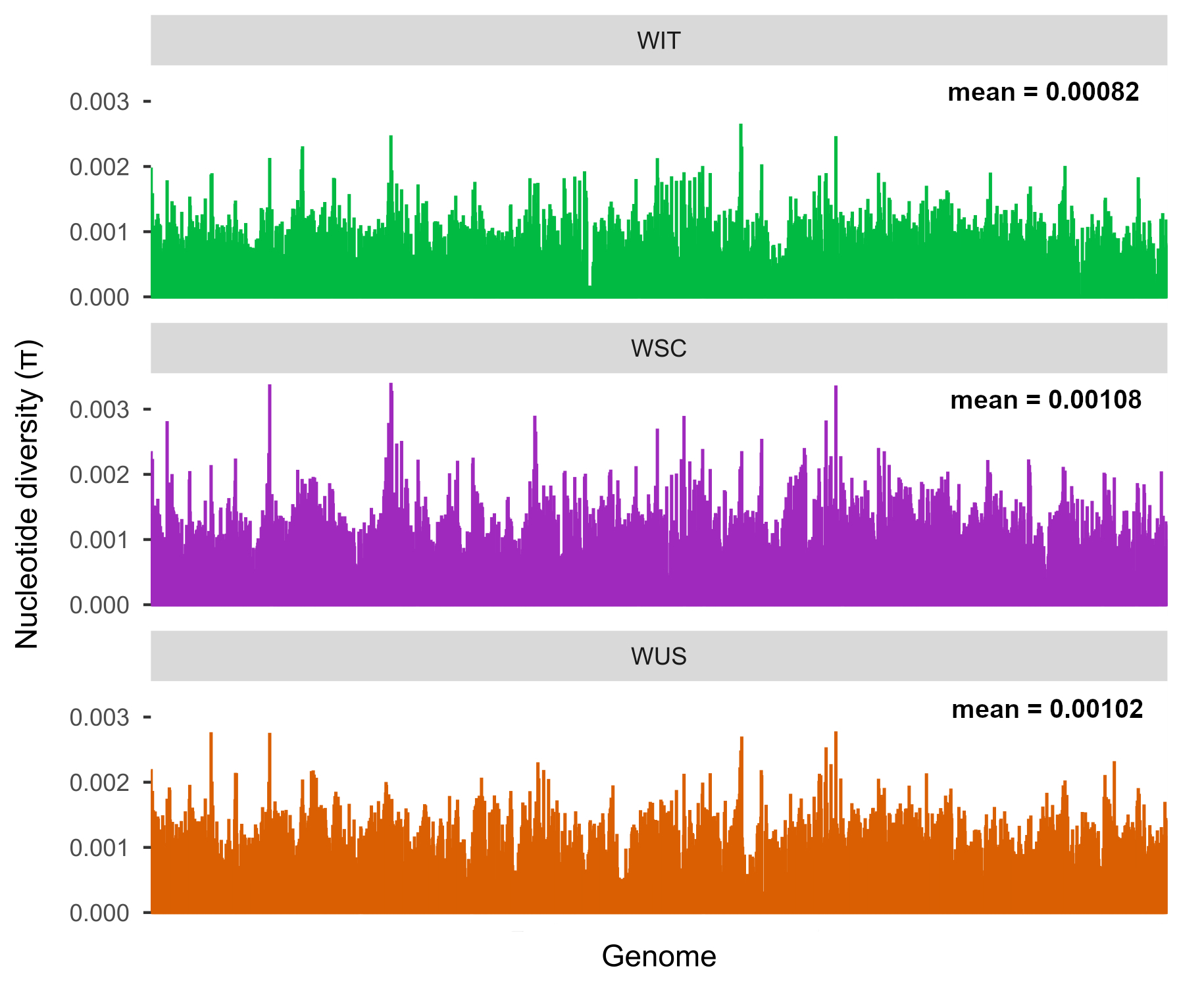

Supplement: esae041_suppl_Supplementary_Data [file esae041_suppl_supplementary_data.zip › esae041_suppl_Supplementary_Figures_1/Supplementary_Fig6.jpeg]

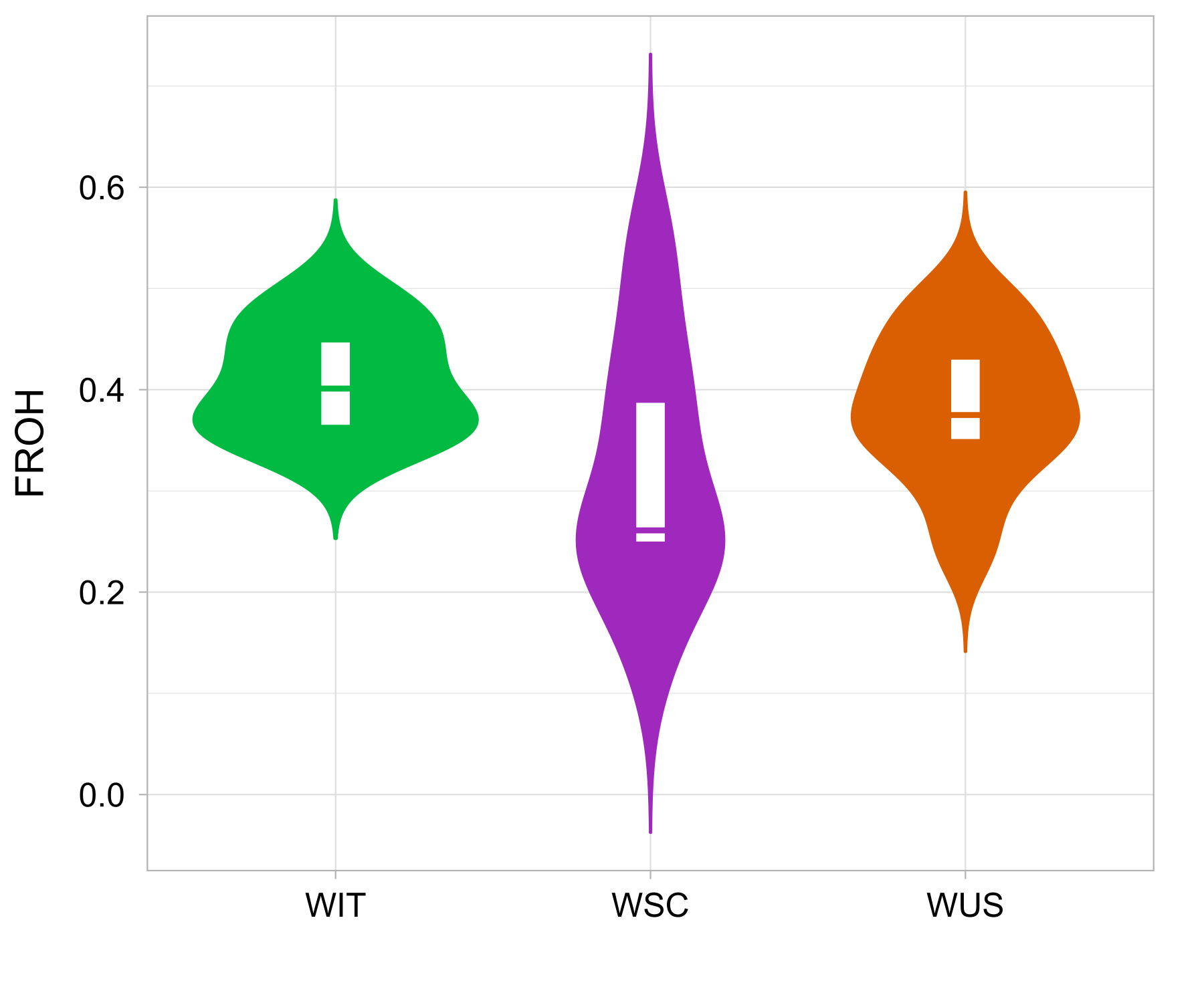

Supplement: esae041_suppl_Supplementary_Data [file esae041_suppl_supplementary_data.zip › esae041_suppl_Supplementary_Figures_1/Supplementary_Fig7.jpeg]

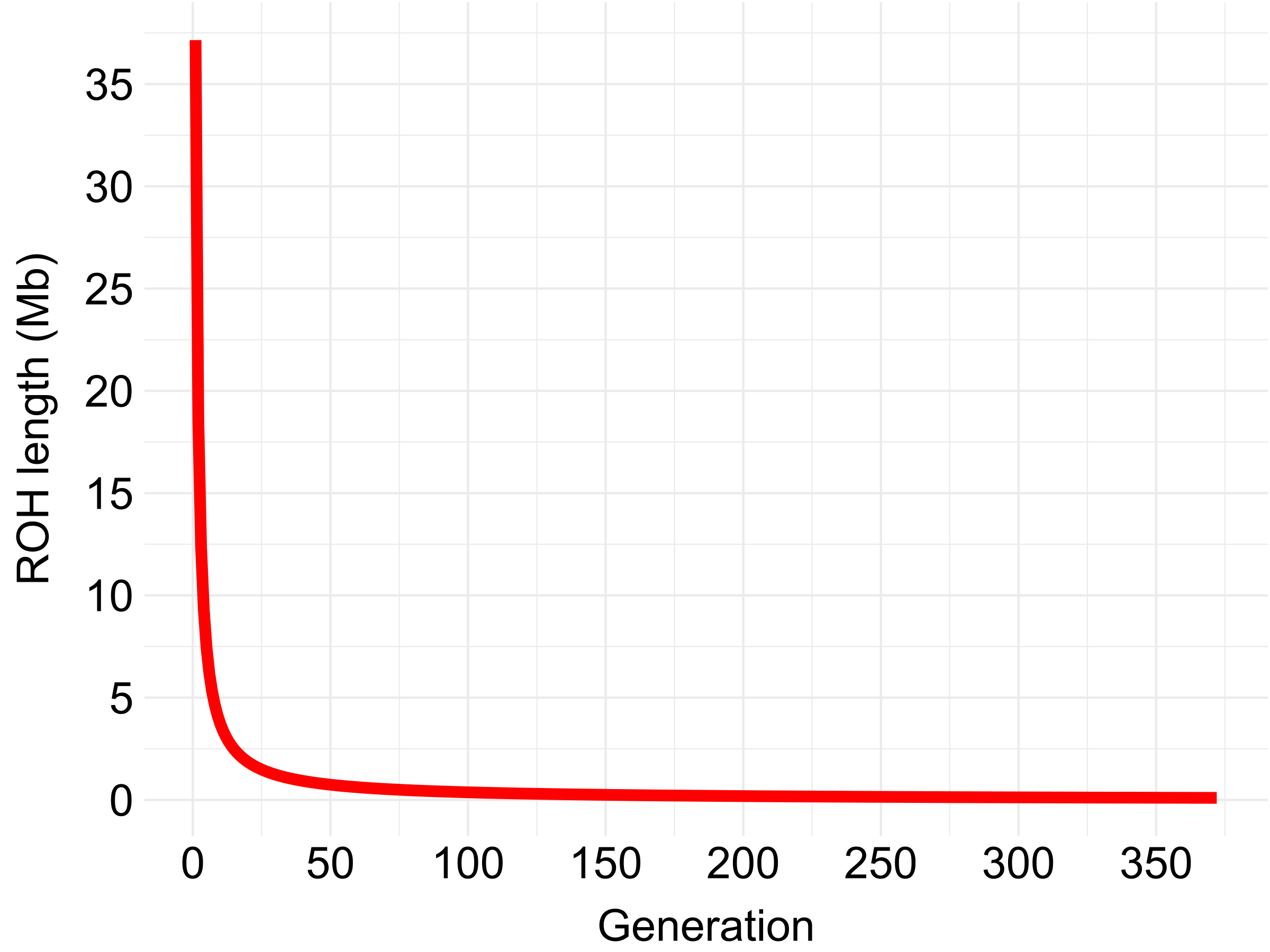

Supplement: esae041_suppl_Supplementary_Data [file esae041_suppl_supplementary_data.zip › esae041_suppl_Supplementary_Figures_1/Supplementary_Fig8.jpeg]

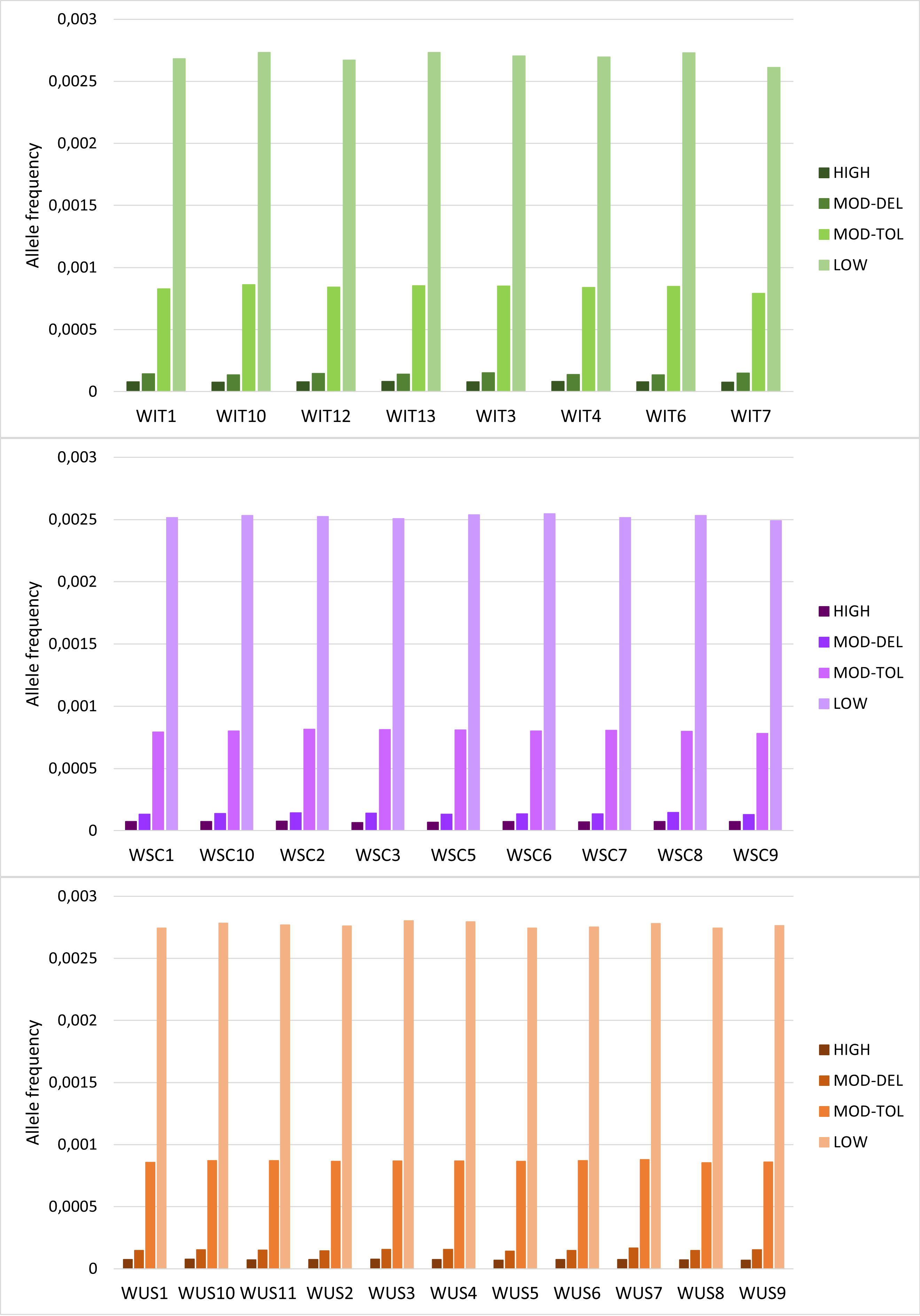

Supplement: esae041_suppl_Supplementary_Data [file esae041_suppl_supplementary_data.zip › esae041_suppl_Supplementary_Figures_1/Supplementary_Fig9.jpeg]
